# Supplementary material for: PAK1 copy number in breast cancer—Associations with proliferation and molecular subtypes
Source: PLoS One. 2023 Jun 27;18(6):e0287608. doi: 10.1371/journal.pone.0287608 (PMC10298784; doi:10.1371/journal.pone.0287608)
Supplement: Attachment — Submitted filename: Response to Reviewers.pdf [file pone.0287608.s001.pdf]

The authors would like to thank both the editor and reviewers for their comprehensive and informative assessment of our manuscript.

Below follows our responses to their comments and suggestions. Page and lines refer to the revised version of the manuscript with track changes.

Responses to editor

Editor's question 1.

Please ensure that your manuscript meets PLOS ONE's style requirements, including those for file naming.

Authors' response:

We have made the necessary changes to the manuscript to comply with the above requirements.

Editor's question 2.

Please provide additional details regarding participant consent. In the ethics statement in the Methods and online submission information, please ensure that you have specified what type you obtained (for instance, written or verbal, and if verbal, how it was documented and witnessed). If your study included minors, state whether you obtained consent from parents or guardians. If the need for consent was waived by the ethics committee, please include this information.

Authors' response:

The Ethics statement has been moved to the Methods section and has been elaborated.

Editor's question 3.

We noticed you have some minor occurrence of overlapping text with the following previous publication(s), which needs to be addressed.

In your revision ensure you cite all your sources (including your own works), and quote or rephrase any duplicated text outside the methods section. Further consideration is dependent on these concerns being addressed.

Authors' response:

Thank you for making us aware of this. The three papers are published by our research group and are thematically related. We have revised the present manuscript rephrasing the text to avoid duplication issues.

Editor's question 4.

Thank you for stating the following financial disclosure:

"The research leading to these results received funding from The Liaison Committee between the Central Norway Regional Health Authority and the Norwegian University of Science and Technology

(NTNU), The Joint Research Committee between St. Olav's Hospital and the Faculty of Medicine and Health Sciences, NTNU (FFU), and the Department of Clinical and Molecular Medicine, NTNU."

Please state what role the funders took in the study. If the funders had no role, please state: "The funders had no role in study design, data collection and analysis, decision to publish, or preparation of the manuscript."

If this statement is not correct you must amend it as needed.

Please include this amended Role of Funder statement in your cover letter; we will change the online submission form on your behalf.

Authors' response:

The statement "The funders had no role in study design, data collection and analysis, decision to publish, or preparation of the manuscript", has now been included in the manuscript under Funding (page 17, lines 19-20).

Editor's question 5.

We note that you have indicated that data from this study are available upon request. PLOS only allows data to be available upon request if there are legal or ethical restrictions on sharing data publicly. For more information on unacceptable data access restrictions, please see http://journals.plos.org/plosone/s/data-availability#loc-unacceptable-data-access-restrictions.

In your revised cover letter, please address the following prompts:

a) If there are ethical or legal restrictions on sharing a de-identified data set, please explain them in detail (e.g., data contain potentially sensitive information, data are owned by a third-party organization, etc.) and who has imposed them (e.g., an ethics committee). Please also provide contact information for a data access committee, ethics committee, or other institutional body to which data requests may be sent.

b) If there are no restrictions, please upload the minimal anonymized data set necessary to replicate your study findings as either Supporting Information files or to a stable, public repository and provide us with the relevant URLs, DOIs, or accession numbers. For a list of acceptable repositories, please see http://journals.plos.org/plosone/s/data-availability#loc-recommended-repositories.

We will update your Data Availability statement on your behalf to reflect the information you provide.

Authors response:

We have amended the Data availability statement to clarify the legislation governing data availability as follows and include the same in the cover letter:

"The background data and data generated in the current study are not publicly available due to General Data Protection Regulations (GDPR), and reasons of personal data protection and sensitivity limitations imposed in the conditions for approval by the Regional Committee for Medical and Health Research Ethics, Midt-Norge (REK 836/2009). However, these data may be made available

from the corresponding author on reasonable request and after approval by the Medical Ethics Committee”.

Editor’s question 6.

We note that you have included the phrase “data not shown” in your manuscript. Unfortunately, this does not meet our data sharing requirements. PLOS does not permit references to inaccessible data. We require that authors provide all relevant data within the paper, Supporting Information files, or in an acceptable, public repository. Please add a citation to support this phrase or upload the data that corresponds with these findings to a stable repository (such as Figshare or Dryad) and provide and URLs, DOIs, or accession numbers that may be used to access these data. Or, if the data are not a core part of the research being presented in your study, we ask that you remove the phrase that refers to these data.

Authors’ response:

In the revised manuscript, we have added this data as Figure 4 and Table 7 in the Results section (page 13, lines 10-11, page 14, lines 1-7).

Editor’s question 7.

Your ethics statement should only appear in the Methods section of your manuscript. If your ethics statement is written in any section besides the Methods, please move it to the Methods section and delete it from any other section. Please ensure that your ethics statement is included in your manuscript, as the ethics statement entered into the online submission form will not be published alongside your manuscript.

Authors’ response:

The Ethics statement has been moved to the Materials and methods section (page 7, lines 17-22) and amended according to Point 5 above.

Editor’s question 8.

Please include your tables as part of your main manuscript and remove the individual files. Please note that supplementary tables (should remain/ be uploaded) as separate "supporting information" files

Authors’ response:

Table 3 is now included in the manuscript (page 9-10). We have not included any supplementary files.

Editor's question 9.

Please review your reference list to ensure that it is complete and correct. If you have cited papers that have been retracted, please include the rationale for doing so in the manuscript text, or remove these references and replace them with relevant current references. Any changes to the reference list should be mentioned in the rebuttal letter that accompanies your revised manuscript. If you need to cite a retracted article, indicate the article's retracted status in the References list and also include a citation and full reference for the retraction notice.

Authors' response:

We have now checked the reference list and it is correct and complete.

Responses to the reviewers:

Reviewer #2:
R2-1. Line 8 (page 4): Patients were diagnosed, however it was not stated if patients were categorized into treatment options. We assumed molecular classification played role in treatment options and this will be important in the effect of PAK 1 and Tamoxifen as mentioned in the discussion.

Authors' response: Information regarding treatment that individual patients received was unavailable to us. The patients included in this study were diagnosed between 1961 and 2008 and would have received treatment according to guidelines available at time of diagnosis. Molecular subtyping was first carried out by our research group and published in 2013 (Engstrom et.al). Molecular subtyping played no role in determining treatment options. We have rephrased to clarify (page 4, lines 15-17). We also discuss this in the Discussion section (page 14, lines 20-23)

R2-2. Table 3 which should highlight the association of PAK 1 CN with Ki67 and Histological grade is missing.

Authors' response: Table 3 is now included in the main manuscript and includes the data mentioned by the reviewer (page 8-9)

R2-3. Kindly check results for line 15 and 16 on page 7 to make sure that comparisons are made with common denominator

Authors' response: This has now been corrected (page 11, line 3)

R2-4. Line 2 of page 7 described described age demography, with inconsistent gap, while table 3 assumed to highlight this data is conspicuously missing.

Authors' response: Table 3 showing patient demography and tumour characteristics according to PAK1 CN was included after the reference list in the original manuscript. It is now included within the main text.

The Cox regression analyses were adjusted for age in five-years categories with the exception of patients ≤ 49 years and ≥ 75. The results are presented in Table 4.

R2-5 The discussions on PAK1 CN and CCND1 was scanty.

Authors' response: We have elaborated this in the Discussion section.

Reviewer #3:

R3-1 Abstract
Aims - change to background or introduction. Define all abbreviations first before use.
Method -This was over summarised. More explanation needed.
Result- Add a comment on how the association was found.
Conclusion- Too long. Let it answer the research question

Authors' response: We have adjusted the Abstract according to the reviewer's recommendations.

R3-2 Body of manuscript
Introduction: Define all abbreviations before use

Authors' response: All abbreviations are now defined at first mention.

R3-3 Material & methods: Check the fish protocol and edit appropriately- Make it flow for ease of understanding.

Authors' response: We have edited the FISH protocol for greater ease of understanding (page 6, lines 3-18).

R3-4 Result: Adequate but need revisions. 'No clear association....' was used several times. It is unclear what it means.

Authors' response: We agree that the term "no clear associations…" is imprecise and have revised the text accordingly (Page 11, lines 4-5, line 8, Page 11, lines 8-9, Page 16, line 23)

R3-5 Pg 7 Line 18-25 and 8:1-3 - revise for clarity; There is repetition of the entire results in the tables- This makes tables/figures redundant. Just mention important findings in-text. Table 3 missing

Authors' response: In this section we have removed the term "no clear association..". Otherwise, we feel that this section presents the most important, but not all, findings in Table 3. We understand that Table 3 was not immediately available in the main text since it was located after the reference section. It is now included in the main text in the revised manuscript.

R3-6 HER2- or HER2 superscript - Stick to one

Authors' response: We have chosen to denote HER2 status using superscript for positive or negative results.

R3-7 Table 5 and 6 confusing. I will suggest the authors stick to the classification stated in the methodology for copy numbers.

Authors' response: In the materials and methods section, we describe the two classifications of *PAK1* copy number used in the study. First, a simple division between no copy number increase (<4) and copy number increase (≥4), and then a three-category division based on the guidelines for classification of *HER2* copy number in breast cancer (<4, ≥4<6, and ≥6). The two methods described are reflected in Tables 5 and 6. The text in the method section has now been modified for greater clarity (page 6, line 24, Page 7, lines 2-3).

R3-8 Discussion: Generally inadequate. Line 4-9: Repetition of result; not necessary.

Authors' response: In the first paragraph of the Discussion part of the manuscript, our intention was to summarize the main findings of the paper. We have rephrased this section reducing its similarity to the Results section (page 14, lines 9-14). The Discussion has been elaborated.

R3-9 Discussion Line 12-19: This was not given attention in the result.

Authors' response: We are uncertain what the reviewer is referring to here. Line 12-19 in the Discussion part of the original manuscript is about strengths and weaknesses of the study. We have elaborated this section for greater clarity regarding histological typing and grading which was carried out by the research group for all cases.

R3-10 No discussion on comparison of primary tumor with corresponding lymph node metastasis

Authors' response: In all, 14 of the 123 cases with corresponding lymph node metastases showed PAK1 CN ≥4 and 5 cases were ≥6. Eight cases showed CN increase (≥4) in both the primary tumour and corresponding lymph node metastases and 6 cases were ≥4 in the primary tumour but not in the metastasis. We have amended the Discussion section accordingly (page 15, lines 18-19)

R3-11 Why was the TMA taken from tutor periphery?

Authors' response: The rationale for selection of tumour periphery in the assembly of the TMAs is now explained on page 16 lines 18-21, and a reference has been added to underline this point.

R3-12 General: The manuscript needs grammar and spelling check.

Authors' response: We have reviewed the manuscript with regard to grammar and spelling.

R3-13 Discussion should be tailored to the results.

Authors' response: Based on comments from all three reviewers the discussion is now better tailored to the results.
